# Co-exposure of cannabinoids with amphetamines and biological, behavioural and health outcomes: a scoping review of animal and human studies

**DOI:** 10.1007/s00213-021-05960-2

**Published:** 2021-10-06

**Authors:** Dimitri Daldegan-Bueno, Lucas O. Maia, Michelle Glass, Didier Jutras-Aswad, Benedikt Fischer

**Affiliations:** 1 Schools of Population Health and Pharmacy, Faculty of Medical and Health Sciences, University of Auckland, 85 Park Rd, Grafton, Auckland 1023 New Zealand; 2 Centre for Applied Research in Mental Health & Addiction, Simon Fraser University, 515 W. Hastings Street,, Vancouver, BC V6B 5K3 Canada; 3 Department of Pharmacology and Toxicology, University of Otago, PO Box 56, Dunedin, 9054 Otago New Zealand; 4 Centre de Recherche, Centre Hospitalier Universitaire de Universite de Montreal (CHUM), 1051 Rue Sanguinet, Montréal, QC H2X 3E4 Canada; 5 Department of Psychiatry and Addiction, Faculty of Medicine, Université de Montréal, Pavillon Roger-Gaudry, 2900 Edouard Montpetit Blvd, Montreal, QC H3T 1J4 Canada; 6 Department of Psychiatry, University of Toronto, 250 College Street, 8Th Floor, Toronto, ON M5T 1R8 Canada; 7 Department of Psychiatry, Federal University of Sao Paulo (UNIFESP), R. Dr. Ovídio Pires de Campos, Sao Paulo, 785 05403-903 Brazil

**Keywords:** Amphetamines, Cannabis, Cannabinoids, Cannabidiol, Co-use, Exposure, Tetrahydrocannabinol, Translational research, Addictive behaviour, Review

## Abstract

**Rationale:**

The growing prevalence of psychostimulant (including amphetamine) use and associated health harms, with limited treatment options, present a global challenge. There is an increasing availability and medical applications of cannabinoids, and growing interest in their therapeutic potential for addictive disorders.

**Objectives:**

The objective of this study is to review available data regarding cannabis/cannabinoid co-use or exposure on amphetamine-related outcomes.

**Methods:**

Towards the present scoping review, we systematically searched four databases (Medline, Web-of-Science, CINAHL Plus and PsycInfo) using cannabis/cannabinoid and amphetamine text-terms identifying peer-reviewed, English-language studies published in 2000–2020 involving multiple methods approaches among both human and animal study samples, assessing the association of co-use/administration of cannabis/cannabinoids products with non-medical amphetamines on biological, behavioural or health outcomes.

**Results:**

Twenty-five articles were included. Pre-clinical studies (*n* = 15) found mostly protective effects of single or repeated cannabinoids administration on rodents in amphetamine addiction models, amphetamine-induced models of human mental disorders (e.g. schizophrenia) and amphetamine-induced neurotoxicity. Human studies (*n* = 10) were more heterogeneously designed (e.g. cross-sectional, case–control, longitudinal) and assessed natural ongoing cannabis and methamphetamine use or dependence, showing mostly enhanced harms in a diversity of outcomes (e.g. mental health, methamphetamine use, cognition).

**Conclusions:**

While human studies suggest cannabis use as an adverse risk factor among non-medical amphetamine users, pre-clinical studies suggest therapeutic potential of cannabinoids, especially cannabidiol, to alleviate amphetamine addiction and harms, including treatment outcomes. Given increasing psychostimulant harms but lack of care options, rigorous, high-quality design studies should aim to translate and investigate pre-clinical study results for potential therapeutic benefits of cannabinoids for amphetamine use/abuse in human subjects.

**Supplementary Information:**

The online version contains supplementary material available at 10.1007/s00213-021-05960-2.

## Introduction

A 0.5% (approximately 27 million people) of the world’s adult population use amphetamines, a central nervous system stimulant. Of these users, approximately 6.6 million (86/100,000 population) are estimated to be dependent, rendering amphetamine substances to be among the most used illicit drugs globally (Degenhardt et al. 2013; Peacock et al. 2018; UNODC 2020). Extra-medical amphetamine use, especially methamphetamine (a more potent structural form (Lake and Quirk 1984)), is associated with elevated risks for both mortality and morbidity outcomes, including acute poisoning, mental health (e.g. depression, psychosis, suicidal behaviours) and neurocognitive impairments, cardiovascular problems, injury and violence (Cumming et al. 2020; Marshall and Werb 2010; Marshall et al. 2011; McKetin et al. 2019; Wood et al. 2006). Amphetamine dependence was responsible for about 0.6% (approximately 326,000 deaths) of global all-cause mortality in 2017, entailing a mortality risk among people with amphetamine dependence up to six times higher than the general population (Farrell et al. 2019). Changes in illegal drug production patterns suggest that amphetamines supply and use have been globally increasing. Global methamphetamine seizures increased by 16% in 2015/16; despite seizures occurring mainly in North America and Asia, a 50% increase of countries reporting seizures has been observed in the last decade. The majority (90%) of dismantled methamphetamine laboratories are located in North America, with the USA harbouring most of these (3,036 or 82%) worldwide in 2017 — mostly supplying the local market (i.e. “kitchen lab”) in contrast with large-scale, export-oriented laboratories in Mexico and East/South-East Asia (Farrell et al. 2019; UNODC 2020).

Currently, there are no pharmacotherapeutic options approved for the treatment of stimulant use disorder, including amphetamines, at any of the main stages of treatment (e.g. withdrawal, relapse prevention) (Brandt et al. 2020; Castells et al. 2016; Morley et al. 2017; Pérez-Mañá et al. 2013; Ronsley et al. 2020; Siefried et al. 2020; Tardelli et al. 2020). The range of evidence-based, non-pharmacotherapeutic treatment or targeted prevention measures is overall highly limited, although select cognitive-behavioural interventions, including contingency management approaches, have shown some promise (Cumming et al. 2016; Fischer et al. 2015a; Knapp et al. 2007; Lee and Rawson 2008; Minozzi et al. 2016; Shearer 2007). This situation underscores a need for the examination of potentially effective therapeutic strategies or interventions for psychostimulant and specifically problematic amphetamine use or use disorders (Fischer et al. 2015b; Kogan and Mechoulam 2007; Shorter et al. 2015). Recently, there has been increased focus on the endocannabinoid system as a possible target for treating drug dependence, craving or withdrawal (Chye et al. 2019; Manzanares et al. 2018; Parsons and Hurd 2015; Spanagel 2020), with a growing number of studies examining the therapeutic effects of cannabinoid agents for addictive disorders (Batalla et al. 2019; Calpe-López et al. 2019; Prud'homme et al. 2015; Turna et al. 2019).

The endocannabinoid system (ECS) is a central biological system composed of at least two endocannabinoids (neurotransmitters; anandamide and 2-arachidoyl glycerol) that bind to cannabinoid receptors expressed throughout the mammalian vertebrate central and peripheral nervous systems. The ECS is centrally involved in regulating essential neural, physiological and cognitive processes, including movement control and motor coordination, learning and memory, mood, stress response and motivation, the immune system, appetite, addictive behaviours and pain modulation, among others (Cabral et al. 2015; Kruk-Slomka et al. 2017; Lu and Mackie 2021; Mechoulam and Parker 2013). The ECS is also involved in key brain structures (e.g. prefrontal, limbic, striatal) and neurotransmitter systems (e.g. serotonin, dopamine) essential in the reward system and drug addiction. Of special interest is the role of CB1 receptors, expressed in brain regions involved in decision-making, withdrawal and relapse, and therefore, essential for modulating the rewarding effects of drugs (Chye et al. 2019; Maldonado et al. 2006; Manzanares et al. 2018; Parsons and Hurd 2015; Spanagel 2020). Moreover, there is increasing evidence and practice of medical usage for some indications (e.g. neuropathic pain, chemotherapy-induced nausea — for reviews see (Abrams 2018; Crippa et al. 2018; NASEM 2017)) and legal availability of cannabinoids products in many countries. Related, a purified cannabis-derived prescription form of cannabinoid has been recently approved in Europe and the USA for selected medical usage indications (GlobeNewswire 2020, 2021; Marcu 2020). Possibly beneficial effects or properties from cannabinoids agents specifically for addictive behaviours related to various substance groups (e.g. opioids, alcohol, nicotine, other stimulants) have been suggested, but no conclusive (e.g. clinical trial-based) evidence or reviews for amphetamine use and related problems presently exist (Abrams 2018; Prud'homme et al. 2015). On this basis, we sought to comprehensively review the existing, multidisciplinary studies and outcome data that have investigated the associations of cannabinoid exposure on amphetamine use and adverse outcomes in different study populations.

## Methods

### Study aim and scope

We conducted a scoping review which aimed to identify and summarize data on possible associations of natural or experimental co-administration of cannabinoids on amphetamine use-related biological, behavioural or health outcomes from different methodological or disciplinary approaches (e.g. pre-clinical, human experimental, observational studies). Cannabinoids were defined to include all-natural cannabis product forms or compositions (e.g. cannabidiol [CBD], tetrahydrocannabinol [THC]) and amphetamine products referred to both amphetamines and the structurally similar methamphetamines. Both cannabinoids and amphetamines were considered irrespective of way of use (e.g. smoke, oral ingestion) or user status (e.g. dependence); while cannabis was considered regardless of the intended purpose of use (e.g. medical, non-medical); only extra-medical use of amphetamines was considered. Considering the over-time evolution in amphetamine-type drug use, types of cannabis products available and the fact that the potential of cannabinoids for addiction-related outcomes has only been investigated more recently (Fischer et al. 2015b), we limited our search to studies published since 2000 with a goal of providing a comprehensive review of relatively recent studies and data.

### Search strategy

The systematic search strategy applied was originally developed for MEDLINE through an iterative process by the co-authors; the final search strategy was revised and adjusted using the Peer Review Electronic Search Strategies (PRESS) checklist (McGowan et al. 2016) and translated to other databases. Four databases were accessed: MEDLINE (PubMed), Web of Science (core collection), CINAHL plus and PsycInfo. The search strategy focused on related cannabis/cannabinoid and amphetamine text words based on MeSH indexing terms (e.g. marijuana, cannabis, CBD, THC, amphetamine, methamphetamine). Given the broad scope and high heterogeneity/interdisciplinarity of study data specifically for outcomes comprised by our scoping review and the intent to comprehensively capture and document these, we did not limit the search by specific outcome terms to avoid a potential restriction, or loss of relevant studies or data. Rather, we left the search for, and identification of, any relevant outcomes as defined by the scope of the review to the subsequent title/abstract and content screening. The full strategy details are presented as supplementary material. Complementary search strategies included comprehensive hand-searching of the reference lists of included studies. The results from the multiple databases were collated and de-duplicated using the Systematic Review Assistant-Deduplication Module (SRA-DM) (Rathbone et al. 2015) and then uploaded to Endnote (v.X9.2), where all screening management tasks were performed.

### Study screening and selection

**Fig. 1:**
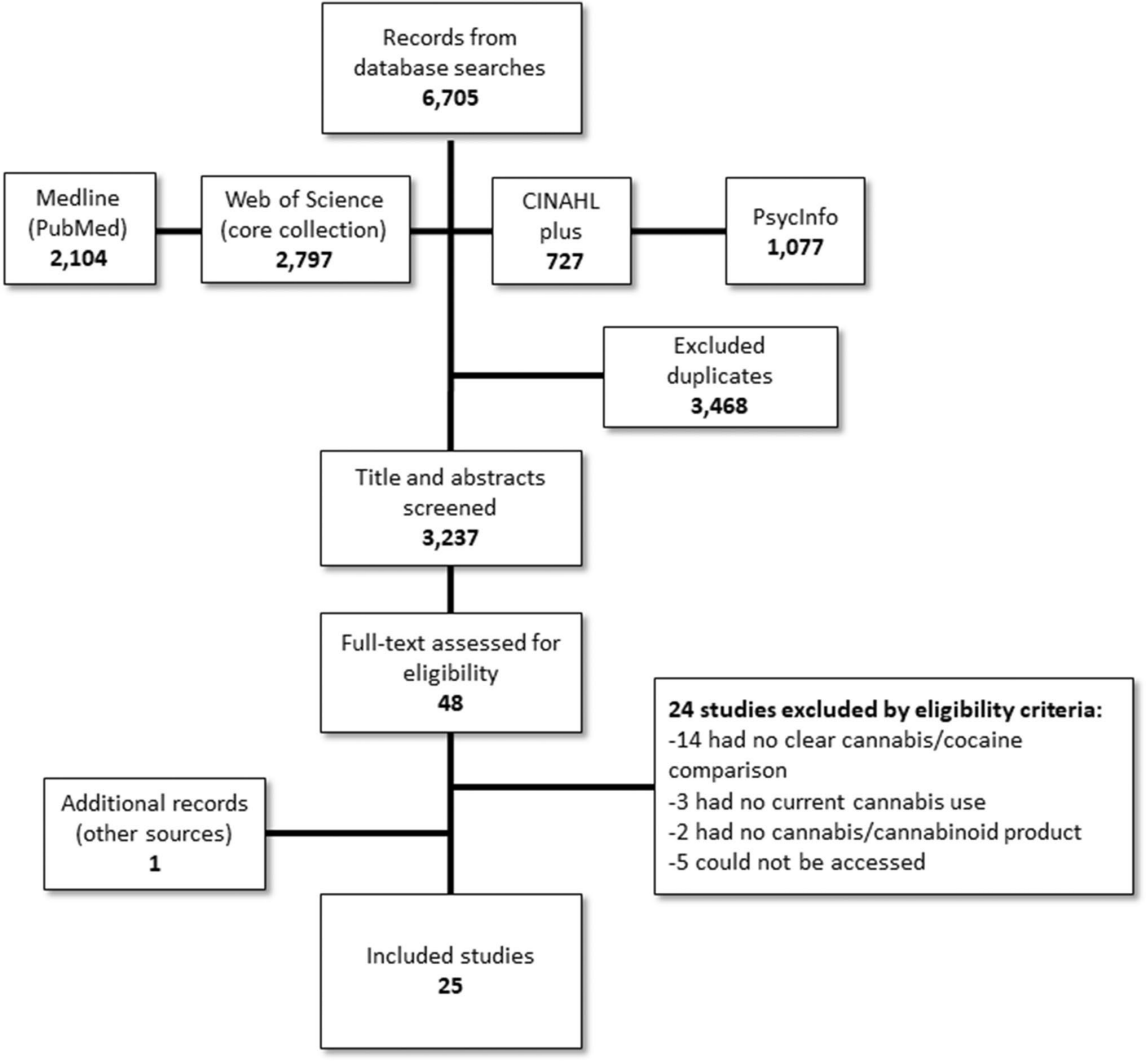
Flowchart of the selection process of studies.

Study screening and selection were based on the following inclusion criteria: (1) original articles based on primary study data; (2) studies examining the direct/immediate, recent (i.e. prior 3 months in humans or same life-phase in animals [e.g. adults, adolescents]), or lifetime (of active users) co-use or co-exposure of cannabinoid products with amphetamine products in human or animal populations; (3) reporting biological, behavioural or health-related measures or outcomes (e.g. brain activity/function, craving, drug use/dependence); (4) with measures or outcomes available that somehow comparatively attributed to amphetamine-only use or groups versus amphetamine and cannabinoid co-use or co-exposure; and (5) in English-language studies published since 2000 to 28 October 2020. Following de-duplication, titles and abstracts were screened by one lead reviewer (DDB), with potentially unclear or ambiguous cases identified and arbitrated towards consensus together with a second reviewer (BF), followed by full-text review of studies identified for possible inclusion by the same procedure. We excluded studies that did not meet the above criteria, as well as reviews, commentaries, case reports and case–control studies that mixed amphetamine- or cannabinoid-related data with other non-target drug use in study groups. To assess the eligibility of case–control studies for inclusion, we verified the exclusive presence of data for amphetamine use with/without cannabinoid use groups and attributable outcomes through information from the studies’ methods and results sections. Abstracts or full studies excluded predominantly did not match the content- or outcome-specific criteria for the review (e.g. no clear cannabis/amphetamine comparison, no current cannabis use) or could not be accessed. The attached flowchart (Fig. 1) presents the main process steps for the present review.

### Data presentation

Given the aim of a comprehensive, scoping review comprising evidence from multi-disciplinary perspectives, both quantitative and qualitative types of studies in different population/samples (e.g. animal, human experimental, treatment, community) were included. For results documentation, we used a descriptive approach, inductively grouping studies principally by type of basic study population and methods/approach, further dividing into study sub-cluster, and narratively summarizing the essential study scope, measures and results. As is common for a scoping review, no risk of bias or quality assessment (Arksey and O'Malley 2005) and no meta-analyses (also due to the heterogeneity of study designs and outcomes) were conducted.

## Results

### Characteristics of included studies

Overall, 6,705 article citations were identified from the database searches which, after deduplication, resulted in a total of 3,237 potentially relevant abstracts that were screened. From these, 48 abstracts were selected for full-text review, with the final 25 articles included in the present review (Fig. 1). Eighteen (> 70%) of the studies were published 2010 onward. Articles broadly ranged across animal-based experimental models (*n* = 15) and human population (*n* = 10) studies. Animal studies involved rodents (rats or mice) experiments of THC or CBD (single or repeat) administration compared to controls (i.e. vehicle administration). On the basis of the data, studies were divided into three clusters: non-contingent models of addiction (e.g. conditioned place preference and sensitization experiments) (*n* = 6), contingent models of addiction (drug self-administration experiments) (*n* = 3) and amphetamine-induced human mental health models and neurotoxicity experiments (*n* = 6). Human population studies comprised methamphetamine and cannabis users or dependents within small or larger population sample. Study designs were cross-sectional (e.g. case–control, experimental intervention), prospective and longitudinal with biological, psycho-behavioural and mental health- or addiction-related outcomes, organized into three topical study clusters: brain structure/metabolism (*n* = 3), neuropsychological function or psychopathology symptoms (*n* = 3) and methamphetamine use patterns (*n* = 4). See Tables 1 and 2 for animal and human studies summaries.

**Table 1:** Summary of pre-clinical studies with cannabinoids and amphetamine co-administration

Study	Sample (sample size)	Cannabinoid	Intervention/design	Outcome measures	Pattern of cannabinoid administration	Main results
**Non-contingent models of addiction**						
Yang et al., 2020	Rats (3–10/group)	CBD 10, 20, 40, and 80 mg/kg, i.p	Effects of CBD exposure on METH-induced conditioned place preference (vehicle-controlled study)	METH-induced conditioned place preference and brain signalling pathways	Single CBD administration during METH-induced conditioning place preference, 1 h before METH administration	↑ CBD reduced METH place preference in a dose-dependent manner (*p* < 0.05) and decreased METH-induced pathways (e.g. Sigma1 receptor, p-AKT, p-GSK-3β, p-CREB) levels in the prefrontal cortex, nucleus accumbens, hippocampus, and ventral tegmental area (*p* < 0.05)
Karimi-Haghighi et al., 2020	Rats (4–5/group)	CBD 10 µg/5 µl, i.c.v	Effects of CBD exposure on neuroinflammatory factors during METH- and stress-induced METH-reinstatement	Neuroinflammatory factors (e.g. IL‐1β, IL‐6, IL‐10, TNF) in the prefrontal cortex and hippocampus	After METH-self administration extinction, single CBD administration 60 min before METH-induced reinstatement in sleep deprived/non-deprived animals	↔ CBD reduced neuroinflammatory factors expression in the prefrontal cortex and hippocampus in the METH-induced reinstatement group (*p* < 0.05), but augmented in the hippocampus of the sleep-deprived group (*p* < 0.05)
Karimi-Haghighi & Haghparast, 2018	Rats (4–6/group)	CBD 10 µg/5 µl i.c.v	Effects of CBD exposure on METH-reinstatement in sleep deprived rats (vehicle-controlled study)	METH-induced reinstatement	After METH-self administration extinction, single CBD administration 60 min before METH–induced reinstatement in sleep deprived/non-deprived animals	↑ CBD reduced METH-induced reinstatement in sleep and non-sleep deprived animals (*F*(3, 22) = 11.13, *p* < 0.001; *F*(2, 15) = 18.29, *p* < 0.001)
Ellgren et al., 2004	Rats (6–8/group)	THC 0.75, 1.5 and 3.0 mg/kg i.p	Effects of THC exposure on AMPH-sensitization in different ages (vehicle-controlled study)	AMPH-sensitization in adolescent and adult rats	Daily THC administration for five days, 7 or 35 days before AMPH administration	↔ THC had no effect on AMPH-sensitization neither in adolescents nor in adults (*F*(3, 97) = 31.2, *p* < 0.001; *F*(4, 95) = 23.2, *p* < 0.001)*
Parker et al., 2004	Rats (8–12/group)	CBD (5 mg/kg) or THC (0.5 mg/kg), i.p	Effects of CBD or THC exposure on the extinction of AMPH place preference memory (vehicle-controlled study)	Extinction of AMPH-induced place preference	Single CBD or THC administration 30 min before METH-induced place preference training	↑ CBD and THC potentiated the extinction of AMPH-induced place-preference learning (F(3, 32) = 4.7, *p* < 0.01)
Lamarque et al., 2001	Rats (9–12/group)	THC 0.6, 3 and 15 mg/kg i.p	Effects of THC exposure on AMPH-sensitization (vehicle-controlled study)	AMPH-sensitization in high- or low-locomotion responsive-animals	Twenty-seven THC administrations in a 3-week period, 3 and 55 days before AMPH administration	↓ THC (0.6 and 15 mg/kg) increased AMPH-induced hyperlocomotion in high-locomotion-responsive animals shortly after treatment (*H*(3) = 7.27, *p* < 0.05) but not after longer time (*p* > 0.05)
**Contingent models of addiction**						
Hay et al., 2018	Rats (10–12/experiment)	CBD 20, 40 and 80 mg/kg, i.p	Effects of CBD exposure on METH-seeking and relapse (vehicle-controlled study)	METH-seeking, relapse and -induced hyperactivity	Single CBD administration 30 min before METH self-administration test or, after extinction, 30 min before METH administration	↑ CBD reduced METH-induced hyperactivity in all doses (F(3, 33) = 11.01, *p* < 0.001); at 80 mg/kg attenuated METH-seeking and relapse (*F*(1, 9) = 13.84, *p* = 0.005; *F*(1.792, 19.707) = 4.96, *p* = 0.021)
Cortright et al., 2011	Rats (6–16/group)	THC 0.4, 0.75, 1.5, 3.0 and 6.0 mg/kg i.p	Effects of THC exposure on AMPH-induced hyperlocomotion and self-administration (vehicle-controlled study)	AMPH-induced hyperlocomotion, self-administration, and nucleus accumbens dopamine levels	Five THC administrations in a 15-day period, 2 days and 2 weeks before AMPH administration or 10 days before AMPH self-administration training	↔ THC (0.75, 1.5, and 3.0 mg/kg) increased AMPH-induced locomotion (*F*(5, 30) = 2.80, *p* < 0.05; *F*(5, 30) = 2.70, *p* < 0.05) but did not affect AMPH self-administration and dopamine levels (data not shown)
Anggadiredja et al., 2004	Rats (4–8/group)	Delta-8-THC 0.32, 1.0 and 3.2 mg/kg i.p	Effect of delta-8-THC exposure on METH-seeking and -reinstatement (vehicle-controlled study)	METH- and cue-induced seeking and reinstatement	Repeated (five days) delta-8-THC administration after METH-extinction session and before METH- or cue-induced reinstatement; single delta-8-THC administration 24 h before METH- or cue-induced reinstatement	↑ Acute delta-8-THC attenuated METH-seeking (0.32 mg/kg, *F*(3, 23) = 13.10, *p* < 0.01), enhanced METH cue-seeking (1 mg/kg, *F*(1,10) = 12.20, *p* < 0.01) and decreased METH- and cue-reinstatement (3.2 mg/kg, *F*(2,19) = 14.6, *p* < 0.0001; *F*(1, 10) = 11.4, *p* < 0.01); at 3.2 mg/kg (repeatedly) decreased METH-reinstatement (*F*(2, 20) = 22.08, *p* < 0.0001)
**Amphetamine-induced mental health model or neurotoxicity**						
Razavi et al., 2020	Rats (5–7/group)	CBD 32 and 160 nmol i.c.v	Effects of CBD on METH-induced memory impairment (vehicle-controlled study)	Locomotor activity, spatial memory (Y-maze), short- and long-term memory (novelty object recognition)	Twice-daily CBD administration for 10 days after 10 days of daily METH administration	↑ CBD had no effect on METH-induced hyperlocomotion (*F*(2, 21) = 0.08; *p* > 0.05) but reversed METH-induced spatial memory, short-term memory and long-term memory impairment (*F*(4, 37) = 7.12; *p* < 0.001; *F*(4, 40) = 3.472; *p* < 0.005; *F*(2, 22) = 5.36; *p* < 0.025)
Renard et al., 2016	Rats (8–10/group)	CBD 100 ng/0.5 ul (per side) i.c	Effects of CBD on D-AMPH-induced schizophrenia model (vehicle-controlled study)	D-AMPH-induced neuronal sensitization and hyperlocomotion; ventral tegmental area molecular pathways	Daily administration for 5 days, right before and 16 days before D-AMPH administration	↑ CBD decreased D-METH-induced hyperlocomotion, dopaminergic neuronal sensitization (*F*(3, 36) = 18.711, *p* < 0.001; *F*(5, 113) = 4.17, *p* < 0.01) and modified ventral tegmental area molecular pathways (e.g. mTOR and p70S6K increase t(6) = − 3.60, *p* < 0.05; t(6) = − 3.70, *p* < 0.05)
Pedrazzi et al., 2015	Mice (8–10/group)	CBD 15, 30 and 60 mg/kg, i.p. or 60 nmol/0.2 μl intra accumbens	Effects of CBD exposure on AMPH-induced PPI disruption (vehicle-controlled study)	AMPH-induced PPI disruption	Single i.p. administration 60 min before PPI test and 30 min before AMPH administration; single intra-accumbens administration 10 min before PPI test and immediately before AMPH administration	↑ Intra-accumbens CBD attenuated amphetamine-induced PPI impairment (*F*(1, 25) = 6.38, *p* = 0.018), while intraperitoneal CBD (30 and 60 mg/kg) was marginally significant (*F*(3, 67) = 4.09, *p* = 0.07)
Castelli et al., 2014	Rats (109 total)	THC 1 and 3 mg/kg i.p	Effects of THC exposure on METH-induced neurotoxicity (vehicle-controlled study)	Body temperature, and neurotoxicity markers in caudate-putamen and cingulate cortex	Multiple administration at several time-points, before or after METH administration	↑ THC decreased caudate-putamen GFAP-IR and nNOS (*F*(2, 32) = 8.12, *p* = 0.001; *F*(2, 37) = 20.53, *p* < 0.001), and cingulate cortex GFAP-IR (*F*(2, 32) = 25.49, *p* < 0.001), but did not affect METH-increased body temperature
Valvassori et al., 2011	Rats (5–15/group)	CBD 15, 30 and 60 mg/kg i.p	CBD effects on a D-AMPH-induced mania model (vehicle-controlled study)	D-AMPH-induced hyperlocomotion, brain damage, and BDNF levels in the hippocampus	Twice a day for 6 (mania reversal) or 14 (mania prevention) days, partially concomitant to D-AMPH administration	↑ CBD did not reverse/prevent D-AMPH-induced hyperlocomotion (data not shown), but protected from D-AMPH-induced brain damage (15 mg/kg) and increased BDNF levels (30 mg/kg) (*p* < 0.05)
Moreira & Guimarães, 2005	Mice (7/group)	CBD 15, 30 and 60 mg/kg i.p	Effects of CBD on D-AMPH-induced hyperlocomotion (vehicle-controlled study)	D-AMPH-induced hyperlocomotion and distance travelled	Single administration 20 min before D-AMPH administration	↑ CBD (30 and 60 mg/kg) reduced D-AMPH-induced hyperlocomotion and distance travelled at selected time points (*F*(45, 132) = 1.49, *p* = 0.044)

^*^Post hoc *p* > 0.05. Legends: Protective (↑), counter-protective (↓) or no effect ( ↔) of cannabis/cannabinoid co-exposure with amphetamine on related outcomes. Abbreviation codes can be found at the abbreviation list

**Table 2:** Summary of studies in humans with cannabis and amphetamine co-use

Study	Sample/groups (sample size)	Participant characteristics	Intervention/design	Outcome measures	Temporality of cannabis use or measure	Main results
**Brain structure/metabolism**						
Sung et al., 2013	METH (9) METH + cannabis (8) Controls (10)	Male/female adolescents with METH and cannabis dependence (criterion not specified), and healthy, non-drug-dependent controls	Case–control study on METH and cannabis neurochemical toxicity	MRS of brain metabolite levels; self-report substance use	Current cannabis dependence diagnostic at admission	↓ METH + cannabis had lower tNAA/PCr + Cr ratio than other groups (F(2, 22) = 4.23, *p* = 0.03) and it negatively correlated with METH and cannabis life-time-doses (F(1, 6) = 11.13, *p* = 0.02; F(1, 6) = 11.44, *p* = 0.02)
Churchwell et al., 2012	METH (9) METH + cannabis (9) Controls (10)	Male/female adolescents with METH and cannabis dependence (DSM-IV) and healthy, non-drug-dependent controls	Case–control study of striatal morphology on METH and cannabis dependents	MRI of striatum volume, novelty-seeking, and lifetime METH use	Current cannabis dependence at admission	↔ METH + cannabis dependents had greater left putamen volume and novelty-seeking score than control (*F*(2, 24) = 4.94, *p* < 0.02; *F*(2, 24) = 3.56, *p* < 0.04); lifetime METH doses correlated to left putamen volume in drug groups (*r*(9) = 0.56, *p* = 0.05; *r*(8) = 0 .65, *p* = 0.04), and with novelty-seeking in METH-only dependents (*r*(9) = 0.64, *p* < 0.03)
Voytek et al., 2005	METH (7) METH + cannabis (7)	Men/women with METH use with/without current cannabis use (< / > 4 joints/month)	Case–control study of brain metabolism on METH and cannabis users	PET of rCMRglc in several brain regions; auditory performance task	Current cannabis use (self-report) at experiment admission	↔ METH + cannabis users had lower rCMRglc in several regions, e.g. right orbitofrontal cortex, temporal gyrus, hippocampus (all *p* < 0.05) but had no between-group difference in auditory accuracy or reaction time (test not shown)
**Neuropsychological function or psychopathology symptoms**						
Cuzen et al., 2015	METH (10) METH + cannabis (10) Controls (20)	Male/female adolescents with METH and cannabis dependence (DSM-IV) and use (> 3 use/week prior 6 months); healthy, non-drug-dependent controls	Longitudinal study of METH and cannabis co-use on neurocognitive functions	Neurocognitive performance and psychopathology scores at baseline and 12-month follow-up	Current cannabis dependence and use (self-report)	↓ METH + cannabis dependents had worse neurocognitive performance, e.g. verbal memory, planning (all *p* < 0.05) than controls, and worse verbal memory (*p* < 0.001) scores than METH-only at baseline but not at follow-up
McKetin et al, 2013	METH (278)	Male/female METH dependents (DSM-IV) with/without cannabis use in the prior month (≥ 16 days)	Longitudinal study of psychotic symptoms on METH dependents	Psychotic symptoms and drug use pattern	Past month cannabis use (self-report) at baseline and 3 follow-ups	↓ METH use was associated with increased odds of experiencing psychotic symptoms (*OR*: 5.3, 95% CI[3.4, 8.3]) and cannabis use (≥ 16 days/last month) further enhanced the risk (95% CI[1.1, 3.5, *p* = 0.02)
Gonzalez et. al., 2004	METH (26) METH + cannabis (27) Controls (41)	Men/women with METH and/or cannabis dependence history (DSM-IV) and healthy, non-drug-dependent controls	Case–control study on neuropsychological performance of METH and cannabis dependents	Neuropsychological performance, comorbid disorders and drug use	Lifetime cannabis dependence at treatment admission	↑ Neuropsychological performance linear worsening: better scores by controls, followed by METH + cannabis and METH-only groups (*F*(1,92) = 9.75, *R*^2^ = 0.096, *p* = 0.002); METH-only had worse scores in learning, recall/retention and general neuropsychological performance than controls (*F*(2, 91) = 3.58, *p* < 0.05); *F*(2, 91) = 4.09, *p* < 0.05; *F*(2, 91) = 4.92, *p* < 0.05); no differences in lifetime comorbid disorders (*p* > 0.05) and METH use (*z* = − 0.28, *p* = 0.78)
**Methamphetamine use patterns**						
Yockey et al., 2020	METH users with/without cannabis use (912)	Men/women with at least one occasion of past-year METH and/or cannabis use	Cross-sectional study on factors correlated to METH use	Demographics and past-year drug use pattern	At least one occurrence of past-year cannabis use (self-report)	↓ Past-year cannabis strongly increased the risk of past-year METH use (*aOR*: 5.51, 95% CI[4.22, 7.19])
Paul et al., 2020	Street-living people (56) Youth-focused care providers (13)	Men/women street-involved drug users	Descriptive qualitative interviews on drug use among street-involved youths	Cannabis use in the context of street entrenchment and drug use trajectories	Natural history	↑ Several participants framed cannabis use as a form to alleviate harms from other drugs, and as being more effective than psychopharmaceutical-assisted treatments for mental health and substance use treatment
Lucas et al., 2013	Medical cannabis dispensary users (404)	Men/women medical cannabis dispensary users	Descriptive survey study on cannabis use as a substitute for other drugs	Cannabis use as a substitution for other drugs use	Cannabis use (self-report) at the time of survey	↑ Cannabis use was reported as a substitute for illicit drugs, including METH (*n* = 7) due to lesser withdrawal, fewer side effects, and better symptom management
Simons et al. 2008	Sample (2,270) containing METH (112) and cannabis (1,087) users	Male/female college students with lifetime (> 1 occasion) and/or current (last 6 month) METH use and lifetime cannabis use	Prospective study on factors associated with METH use	Demographic and drug use pattern at baseline and 6-month follow-up	Lifetime cannabis use at baseline (self-report)	↓ Multivariate path-analysis model found METH-lifetime use at baseline and METH use at follow-up to be positively associated with lifetime cannabis use (*b* = 1.03, *p* < 0.001; *b* = 0.60, *p* < 0.001)

Legends: Protective (↑), counter-protective (↓) or no effect ( ↔) of cannabis/cannabinoid co-use with amphetamines on related outcomes. Abbreviation codes can be found at the abbreviation list

### Pre-clinical studies in animals

#### Non-contingent models of addiction (n = 6)

Four vehicle-controlled studies investigated the effects of single CBD or THC administration in rats with an amphetamine- or methamphetamine-conditioned place preference (i.e. preference to a drug-paired environment) on amphetamine-reinstatement (i.e. return of drug-seeking after extinction of drug-seeking behaviour) and neurobiological outcomes. THC (0.5 mg/kg, i.p.) or CBD (5 mg/kg, i.p.) administration 30 min before amphetamine place preference extinction trials (*n* = 8–12/group) potentiated the extinction of amphetamine-induced place-preference (*F*(3, 32) = 4.7, *p* < 0.01, post hoc *p* < 0.05) (Parker et al. 2004). CBD (10, 20, 40 and 80 mg/kg, i.p.) administrated 1 h before methamphetamine (2 mg/kg, i.p.) administration (*n* = 3–10/group) reduced methamphetamine-induced place preference in a dose-dependent manner (*p* < 0.05) and decreased several methamphetamine-induced signalling pathways (e.g. Sigma1 receptor, p-AKT, p-GSK-3β, p-CREB) levels in the prefrontal cortex, nucleus accumbens, hippocampus, and ventral tegmental area (all *p* < 0.05) (Yang et al. 2020). Single intracerebral ventricular CBD (10 µg/5 µl) administration 60 min before methamphetamine (0.25 and 0.5 mg/kg, s.c.) administration in animals with/without sleep deprivation stress and methamphetamine-induced conditioned place preference (*n* = 4–6/group) impaired methamphetamine-reinstatement response in both sleep and non-sleep deprived animals (*F*(3, 22) = 11.13, *p* < 0.001; *F*(2, 15) = 18.29, *p* < 0.001, both post hoc *p* < 0.05) (Karimi-Haghighi and Haghparast 2018). In a sequencing study with the same experimental conditions (*n* = 4–5/group), IL‐1β, IL‐6 and IL‐10 mRNA expression in the prefrontal cortex, and TNF‐α, IL‐1β and IL‐6 in the hippocampus were reduced in methamphetamine-induced reinstatement animals (*p* < 0.05). TNF‐α, IL‐1β, IL‐6 and IL‐10 mRNA expression levels were augmented in the hippocampus, but IL‐10 was reduced in the prefrontal cortex of sleep-deprivation-induced reinstatement animals (*p* < 0.05) (Karimi-Haghighi et al. 2020).

Two vehicle-controlled studies in rats assessed the effects of repeated THC administration on amphetamine-induced sensitization (i.e. a drug-dependent increase of locomotor activity). Repeated THC (0.75, 1.5 and 3.0 mg/kg, i.p.) administration for 5 days (*n* = 6–8/group) had no effect on amphetamine-induced sensitization (0.5 and 2.0 mg/kg, i.p.) in adolescent or adult rats (*F*(3, 97) = 0.38, *p* < 0.001; *F*(4, 95) = 23.2, *p* < 0.001, post hoc *p* > 0.05) (Ellgren et al. 2004); however, 27 THC (0.6, 3 and 15 mg/kg, i.p.) administrations in a 3-week period (*n* = 9–12/group), 3 and 55 days before amphetamine (1 mg/kg, i.p.) administration, increased amphetamine sensitization in previously high-locomotion-responsive animals shortly after treatment (*H*(3) = 7.27, *p* < 0.05, post hoc *p* < 0.05), but not after longer time (*p* > 0.05) (Lamarque et al. 2001).

Overall, studies with non-contingent models of amphetamine/methamphetamine exposure showed a protective potential of CBD by reducing amphetamine reward and risk of relapse and facilitating related memory extinction. CBD was also shown to reduce neuroinflammatory factors in the brain, though it also enhanced the same factors in one specific (sleep-deprivation stress) condition. While THC was found to potentiate the extinction of amphetamine-related memory, it also showed no or counter-protective effects, specifically in vulnerable drug animals, as measured by methamphetamine sensitization.

#### Contingent models of addiction (n = 3)

Three vehicle-controlled studies assessed the effects of single or repeated CBD or THC administration in rats trained to self-administer amphetamine or methamphetamine on cue- or amphetamine/methamphetamine-seeking (response to acquire drug), relapse (drug-seeking after drug self-administration extinction) or reinstatement. Single CBD (20, 40 and 80 mg/kg, i.p.) administration in animals trained to self-administer methamphetamine (0.05 ml/infusion, i.v.) 30 min before methamphetamine-seeking and relapse trials (*n* = 10–12/experiment) attenuated, at 80 mg/kg, methamphetamine-seeking and relapse responses (*F*(1, 9) = 13.84, *p* = 0.005; *F*(1.792, 19.707) = 45.96, *p* = 0.021 both post hoc *p* < 0.05), and at all doses reduced methamphetamine-induced hyperactivity (*F*(3, 33) = 11.01, *p* < 0.001, all post hoc *p* < 0 0.05) (Hay et al. 2018). Acute delta-8-THC (0.32, 1.0 and 3.2 mg/kg, i.p.) administration before methamphetamine self-administration extinction sessions (*n* = 4–8/group) attenuated, at 0.32 mg/kg, methamphetamine-seeking (*F*(3, 23) = 13.10, *p* < 0.01, post hoc *p* < 0.001), at 1 mg/kg enhanced methamphetamine cue-seeking (*F*(1, 10) = 12.20, *p* < 0.01, post hoc *p* < 0.001) and at 3.2 mg/kg decreased methamphetamine- and cue-reinstatement (*F*(2, 19) = 14.6, *p* < 0.0001; *F*(1, 10) = 11.4, *p* < 0.01, both post hoc *p* < 0.01), while repeated delta-8-THC (5 days, 3.2 mg/kg, i.p.) administration decreased methamphetamine-reinstatement (*F*(2, 20) = 22.08, *p* < 0.0001, post hoc *p* < 0.001) (Anggadiredja et al. 2004). However, five administrations of THC (0.4, 0.75, 1.5, 3.0 and 6.0 mg/kg i.p.) in a 15-day period before amphetamine administration (0.75 mg/kg, i.p.) in animals with amphetamine self-administration history (*n* = 6–12/group) had no effect on dopamine levels and amphetamine self-administration (data not shown), but increased amphetamine-sensitization (*F*(5, 30) = 2.80, *p* < 0.05; *F*(5, 30) = 2.70, *p* < 0.05, both post hoc *p* < 0.05) (Cortright et al. 2011).

Overall, contingent models of amphetamine/methamphetamine addiction studies generally showed a relapse-preventive potential of CBD and THC by reducing the motivation to seek and consume methamphetamine, while one study showed THC to increase amphetamine-induced hyperlocomotion but without affecting its self-administration.

### Amphetamine-induced mental health model or neurotoxicity (n = 6)

Four vehicle-controlled studies assessed the effects of single or repeated CBD administration, either by intraperitoneal or intra-nucleus accumbens injections, before (D-) amphetamine administration in a model of human psychoses/mental health (e.g. schizophrenia, bipolar disorder) on behavioural and neurobiological outcomes. Single CBD (15, 30 and 60 mg/kg, i.p.) administration in mice (*n* = 7/group) 20 min before D-amphetamine administration (5 mg/kg, i.p.), at 30 and 60 mg/kg, reduced D-amphetamine-induced hyperlocomotion and total distance travelled at selected time-points (*F*(45, 132) = 1.49, *p* = 0.044, post hoc *p* < 0.05) (Moreira and Guimarães 2005). Single CBD (60 nmol/0.2 μl, intra-accumbens) administration in mice (*n* = 8–10/group) immediately before amphetamine (10, 30 and 60 mg/kg, i.p.) administration and 10–30 min before pre-pulse inhibition test (i.e. reflex response inhibition by a weak sensory event) attenuated amphetamine-induced pre-pulse impairment (*F*(1, 25) = 6.38, *p* = 0.018, post hoc *p* < 0.05), but this effect was only marginally significant at 30 and 60 mg/kg doses (*F*(3, 67) = 4.09, *p* = 0.07, post hoc *p* < 0.05) (Pedrazzi et al. 2015). Repeated CBD (100 ng/0.5 ul, intra-accumbens) for five or 16 days and (in another group) immediately before a D-amphetamine-induced (100 ng/0.5 ul, intra-accumbens) model of schizophrenia in rats (*n* = 8–10/group) decreased D-amphetamine-induced hyperlocomotion, dopaminergic neuronal sensitization (*F*(3, 36) = 18.711, *p* < 0.001; *F*(5, 113) = 4.17, *p* < 0.01, both post hoc *p* < 0.05) and modified ventral tegmental area molecular pathways (e.g. mTOR and p70S6K increase *t*(6) =  − 3.60, *p* < 0.05; t(6) =  − 3.70, *p* < 0.05) (Renard et al. 2016). However, twice-daily CBD (15, 30 and 60 mg/kg, i.p.) administration for 6 or 14 days (*n* = 5/group), partially concomitant to repeated D-amphetamine (2 mg/kg, i.p.) administration, did not reverse or prevent a bipolar manic model (i.e. D-amphetamine-induced hyperlocomotion) (data not shown), but at 15 mg/kg protected from the D-amphetamine-induced brain damage and at 30 mg/kg increased BDNF levels in the hippocampus (*p* < 0.05) (Valvassori et al. 2011).

Two studies investigated the effect of THC or CBD administration on methamphetamine-induced neurotoxicity on behavioural or biological outcomes of rats. Single THC (1 and 3 mg/kg, i.p.) administration before or after methamphetamine-induced (10 mg/kg, s.c.) neurotoxicity (total *n* = 109) decreased caudate-putamen GFAP-IR and nNOS-positive neurons in selected doses (*F*(2, 32) = 8.12, *p* = 0.001; *F*(2, 37) = 20.53, *p* < 0.001, both post hoc *p* < 0.01) and in all doses decreased cingulate cortex GFAP-IR (*F*(2, 32) = 25.49, *p* < 0.001, post hoc *p* < 0.001) but did not affect METH-increased body temperature (Castelli et al. 2014). Repeated CBD administration (32 and 160 nmol i.c.v./day) during a 10-day methamphetamine-abstinence period did not affect methamphetamine-induced hyperlocomotion (*F*(2, 21) = 0.08; *p* > 0.05), but both doses reversed methamphetamine-induced spatial memory impairment (*F*(4, 37) = 7.12; *p* < 0.001, post hoc *p* < 0.05), short-term memory impairment (*F*(4, 40) = 3.472; *p* < 0.005; post hoc *p* < 0.05) and at 160 nmol, long-term memory impairment (*F*(2, 22) = 5 0.36; *p* < 0.025, post hoc *p* < 0.05) (Razavi et al. 2020).

Overall, for amphetamine-induced mental health, there was a CBD-related protective effect for amphetamine/methamphetamine-induced animal models of psychosis but not mania, suggesting antipsychotic-like action. Also, CBD and THC protected against amphetamine/methamphetamine-induced brain damage.

## Studies in humans

### Brain structure/metabolism (n = 3)

Two case–control studies compared adolescents with methamphetamine dependence, methamphetamine and cannabis dependence and healthy controls on their brain structure or metabolism. Using proton magnetic resonance spectroscopy (MRS) to investigate neurochemical toxicity in the cingulate brain area in South African adolescents (*n* = 8–10/group), Sung et al. (2013) found no between-group differences in mI/PCr + Cr, GPC + PC/PCr + Cr and Glu + Gln/PCr + Cr metabolite (*p* > 0.05), but methamphetamine and cannabis dependents (criteria not specified) had lower tNAA/PCr + Cr ratio than methamphetamine-only dependent subjects and healthy, non-drug-dependent controls (*F*(2, 22) = 4.23, *p* = 0.03, post hoc *p* < 0.05). Also, tNAA/PCr + Cr ratio correlated with several drug use patterns, e.g. a negative correlation with methamphetamine and cannabis life-time-doses (*F*(1, 6) = 11.13, *p* = 0.02; *F*(1, 6) = 11.44, *p* = 0.02). Churchwell et al. (2012) used structural magnetic resonance images (MRI) and found methamphetamine- and cannabis-dependent (DSM-IV) US-based adolescents (*n* = 9–10/group) to have greater left putamen volume and higher novelty-seeking scores than healthy, non-drug-dependent controls (*F*(2, 24) = 4.94, *p* < 0.02; *F*(2, 24) = 3.56, *p* < 0.04, both post hoc *p* < 0.05); also, lifetime methamphetamine doses positively correlated to left putamen volume in methamphetamine and methamphetamine + cannabis dependents (*r*(9) = 0.56, *p* = 0.05; *r*(8) = 0.65, *p* = 0.04), and with novelty-seeking in methamphetamine-only dependents (*r*(9) = 0.64, *p* < 0.03).

Another study, using positron emission tomographic (PET) in US-based methamphetamine users with/without cannabis co-use (> 4 joints/month, *n* = 7/group) during an auditory performance task (i.e. discrimination of high-pitched tone among lower-pitched tone distracters) found methamphetamine + cannabis users to have lower cerebral glucose metabolism (rCMRglc) in several brain regions, e.g. right orbitofrontal cortex, temporal gyrus, hippocampus (all *p* < 0.05) but no difference in auditory performance accuracy (*mean* ± *SEM* [log 1 + % errors]): 0.69 ± 0.16; 0.80 ± 0.10) or reaction time (0.65 ± 0.06; 0.75 ± 0.07) was found (Voytek et al. 2005).

Overall, brain structure and metabolism studies showed that cannabinoids co-use with methamphetamines is associated with neurotoxicity and abnormal brain function.

### Neuropsychological function or psychopathology symptoms (n = 3)

Two case–control studies assessed neuropsychological performance in adolescents or adults with methamphetamine dependence, methamphetamine + cannabis dependence and healthy controls. One study among South African adolescents with methamphetamine + cannabis dependence (DSM-IV) (*n* = 10) found their performance to be impaired in several neuropsychological tasks (e.g. verbal memory, planning, all *p* < 0.05) compared to healthy, non-drug-dependent controls (*n* = 20), and worse verbal memory (*p* < 0.001) compared to methamphetamine-only dependents (*n* = 10) (Cuzen et al. 2015). Conversely, Gonzalez et al. (2004) reported a linear worsening of neuropsychological performance in a US-based adult sample when considering lifetime cannabis/methamphetamine dependence (DSM-IV), with better scores in the control (i.e. healthy, non-drug dependents) group (*n* = 41), followed by methamphetamine + cannabis (*n* = 27) and methamphetamine-only dependents (*n* = 26) groups (*F*(1, 92) = 9.75, *R*^2^ = 0.096, *p* = 0.002). Moreover, methamphetamine-only dependents had worse learning, recall/retention and general neuropsychological performance scores than controls (*F*(2, 91) = 3.58, *p* < 0.05); *F*(2, 91) = 4.09, *p* < 0.05; *F*(2, 91) = 4.92, *p* < 0.05, all post hoc *p* < 0.05). This same study also did not find between-group differences in lifetime psychiatric comorbid disorders (*p* > 0.05) and methamphetamine use among groups (*z* =  − 0.28, *p* = 0.78).

In regard to psychopathology, an Australian-based longitudinal prospective cohort with methamphetamine dependents (DSM-IV, *n* = 278) found prior-month methamphetamine use to be associated with increased odds of experiencing psychotic symptoms (*OR*: 5.3, 95% CI [3.4, 8.3]) and prior-month cannabis use (≥ 16 days) to further increase the odds of experiencing psychotic symptoms (95% CI [1.1, 3.5], *p* = 0.02), even when controlling for alcohol use (McKetin et al. 2013).

Overall, two studies suggested that cannabis co-use was associated with increased methamphetamine-related harm by increasing neurocognitive impairment and psychotic symptoms, while one found no exacerbation of cannabis exposure on methamphetamine-related neurotoxicity measures.

### Methamphetamine use patterns (n = 4)

Two US-based studies assessed different factors associated with methamphetamine use, including cannabis use. A cross-sectional study with 18–34-year-old methamphetamine-users (at least 1 prior-year use instance) using data from the population-representative National Survey of Drug Use and Health (NSDUH, 2015/18) found that self-reported past-year cannabis use strongly increased the risk of past-year methamphetamine use (*n* = 912) (*aOR*: 5.51, 95% CI [4.22, 7.19]) (Yockey et al. 2020). A prospective study among a convenience sample of rural area college students (18–25 years old), based on a self-report electronic questionnaire containing methamphetamine (*n* = 112) and cannabis (*n* = 1,087) users, found methamphetamine-lifetime use at baseline and methamphetamine-use at 6-month follow-up (controlled by lifetime-methamphetamine use) to be positively associated with lifetime cannabis use (*b* = 1.03, *p* < 0.001; *b* = 0.60, *p* < 0.001), although 89% of cannabis users reported having never used methamphetamine at 6-month follow-up (Simons et al. 2008).

Two Canada-based descriptive studies explored the influence of cannabis use on the use of other drugs (including methamphetamine) from the participants’ perspectives. A study with anonymous self-reported survey-data from a sample of medical cannabis dispensary users (*n* = 404) reported cannabis use as a substitute for illicit drugs (36.1%), including methamphetamine (*n* = 7), due to perceived lesser withdrawal, fewer side effects and better symptom management characteristics ascribed to cannabis use (Lucas et al. 2013). In-depth qualitative interviews with street-involved young people (16–26 years old, *n* = 56) and youth-focused care providers (*n* = 13) reported that most drug users engaged in daily cannabis use at the same time as they used other substances (including methamphetamine), and several participants described cannabis use as a form to alleviate harms from other drugs’ use. Respondents also perceived cannabis use as being more effective than psychopharmaceutical-assisted treatments for mental health and substance use treatment, and few participants reported harms related to the intensive-cannabis use (Paul et al. 2020).

Overall, two studies on methamphetamine use patterns showed cannabis use to be associated with increased methamphetamine use; conversely, two self-reports suggested cannabis use to alleviate harms from, or to substitute for, methamphetamine use.

## Discussion

Animal studies retrieved in this review showed mostly protective effects of both CBD and THC upon amphetamine exposure in models used to investigate several phases of amphetamine addiction (e.g. drug-seeking, relapse, extinction) (Kuhn et al. 2019; Spanagel 2017), and neurobiological markers (e.g. neurotoxicity, brain damage). While almost all CBD-based studies disclosed protective results, including for alleviating amphetamine-induced psychosis, some studies with THC-agents reported no effect and one showed a counter-protective effect, suggesting a facilitated progression of THC towards amphetamine use in vulnerable individuals (i.e. prior high-locomotion responsive animals).

The endocannabinoid system has been a topic of growing interest as a possible target for treating craving and addiction given its relationship to reward pathways, and substances acting on this system have been suggested as possible candidates for the pharmacotherapeutic treatment of substance use disorders (Batalla et al. 2019; Chye et al. 2019; Manzanares et al. 2018; Parsons and Hurd 2015; Prud'homme et al. 2015; Spanagel 2020). Although the full mechanisms of action of CBD are yet to be determined, CBD has been reported to act as a negative allosteric modulator of the CB_1_ receptor and is unlikely to produce a rewarding effect (e.g. voluntary self-administration) (Ibeas Bih et al. 2015; Laprairie et al. 2015; Spanagel 2020). CBD also exerts its actions through other circuitry relevant to addictive disorder (e.g. 5HT1a, α7-nicotinic receptors), and some of its pharmacological effects such as reversing amphetamine-induced neuroinflammation and cognitive deficits, as well as alleviating mental disorder comorbidities (e.g. anxiety, depression) can be implied as a therapeutic benefit for amphetamine addiction (Calpe-López et al. 2019; Manzanares et al. 2018). In contrast, THC features substantial differences in its pharmacological actions that may account for the more heterogeneous results. By acting as an agonist of the CB_1_ receptor, THC produces a dose-sensitive rewarding effect, with increasing doses shifting to negative, instead of positive, reinforcement (Spanagel 2020). Moreover, the differences in results observed in THC studies, ranging from protective to counter-protective effects, indicate a possible benefit from THC to amphetamine addiction that may depend on experimental factors (e.g. dose, length and timing of administration).

Overall, pre-clinical studies identified in this review aggregate evidence that phyto-cannabinoids, especially CBD, may have protective potential both for amphetamine addiction in several phases (e.g. relapse, abstinence) and for other amphetamine-related consequences (e.g. neurotoxicity). Also, CBD showed some protective effects for amphetamine-induced models of psychosis. Amphetamine-induced sensitization is a useful animal model for assessing human mental health conditions (e.g. schizophrenia, mania) that relies on the behavioural and neurochemical, including toxic, effects of amphetamine (Featherstone et al. 2007; Young et al. 2011). Moreover, investigating CBD’s potential on the amphetamine-induced model of mental health can provide insights  into behavioural and neuroprotective factors that may be used translationally not only for specific mental illness populations, but also to amphetamine users. For instance, some of the known psychiatric (e.g. acute mania or psychotic) symptoms of amphetamine exposure observed in different populations (Anand et al. 2000; Curran et al. 2004; McKetin et al. 2013; Strakowski and Sax 1998) could be reduced by CBD-related actions.

In contrast, studies in human mostly showed counter-protective effects or no effect on the co-use of cannabis with amphetamine for several outcomes, including brain (e.g. MRS, MRI), mental health, cognition (e.g. neuropsychological performance, psychotic symptoms) and amphetamine use. Several factors may account for the observed discrepancies with the human-based results and the growing body of literature suggesting a therapeutic potential of cannabinoids for addictive, including psychostimulant- and amphetamine-related behaviours (Abrams 2018; Prud'homme et al. 2015). First, these studies are highly heterogeneous, with fundamental differences in design (e.g. case–control, longitudinal, prospective), cannabis use pattern (e.g. use or dependence), outcomes measured and population studied (e.g. college students, drug dependents). One major difference between the pre-clinical and human studies that may account to the discrepant results is the cannabinoid/cannabis exposure pattern. While all human studies assessed cannabis and amphetamines co-used within a naturalistic form of use and/or dependence, with no studies assessing the direct administration (e.g. experimental setting) of cannabis or cannabis products (e.g. CBD, THC, Sativex®) on amphetamine users, pre-clinical studies did not evaluate cannabinoids within an abuse model (e.g. THC self-administration learning, conditioned place preference).

Given the non-clinical nature, the human studies included did not control other relevant factors on addiction, such as drug (cannabinoid)-related conditioned response (e.g. eliciting drug desire, triggering relapse) and social interactions (Bardo et al. 2013; Kenny et al. 2003; O'Brien et al. 1998; Pelloux et al. 2019). In addition to the common pathway (i.e. inhalation) that cannabis and amphetamine use may share, the ongoing, naturalistic use of cannabis by participants, often taken as co-dependence, may entail a poly-substance use scenario, which is commonly associated with more co-occurring and/or severe problems (e.g. psychological distress, anxiety, depression) among users (Connor et al. 2013, 2014). Also, it could be possible that amphetamine users with co-morbid conditions use cannabis in an attempt to self-regulate or self-treat comorbid mental health or cognitive problems experienced. Some qualitative reports, mostly focusing on crack cocaine but also methamphetamine use, highlight intentional cannabis use patterns towards alleviation of psychostimulant-related problems (Andrade et al. 2011; Gonçalves and Nappo 2015; Paul et al. 2020).

On this basis, the absence of more controlled studies (e.g. clinical trials) for psychostimulants, and for amphetamines specifically, still represents a major gap in the field; conducting such studies would allow for a better translation of the pre-clinical findings into human/clinical setting. Despite these translational limitations, some of the human studies in this review suggest that cannabis use, especially when involving subjects with substance use disorder/dependence, may entail increased risks for adverse outcomes or co-morbidities among users, which highlights the importance of considering potential cannabis use-related risks as much as its therapeutic potential among amphetamine users.

Two descriptive studies in human samples reported cannabis use reducing amphetamine use-related harms and detrimental effects, including one qualitative in-depth self-report study. Similar results have been reported more frequently for the co-use of cannabis with crack cocaine, and some observers have already proposed for cannabis application use a pragmatic, substitutive or therapeutic option for other harmful drug use to be further investigated (Andrade et al. 2011; Gonçalves and Nappo 2015; Lau et al. 2015; Socías et al. 2017). Different dynamics in the context of drug use, motivational aspects or subpopulation specificities could be involved in these contrasting, protective results. However, while descriptive designs with qualitative approaches are relevant to investigate specific populations (Patton 2015), the identified studies are limited in nature since they rely on participants’ (or others’) subjective perception/beliefs and lack control measures for generalization (e.g. control group, standardized measures, biased sample), and, as such, conclusions are limited. At best, these results, together with the mostly enhanced harm outcomes in human study populations described, suggest that any possible benefits from cannabis use regarding amphetamines addiction is likely strongly dependent on patterns of cannabis use (e.g. timing, motivation, dependence).

Although the limitations of the studies in humans showing some protective effects related to cannabinoids use (i.e. less frequently and/or less controlled than studies finding counter-protective effects), they reinforce, together with the pre-clinical studies’ results, the necessity to conduct controlled studies on cannabinoids and their potential to alleviate amphetamine addiction or amphetamine-related deficits. Overall, in addition (or due) to the non-specific cannabinoid’s, especially CBD, mechanisms, the therapeutic potential for substance use disorder may crucially vary according to the substance. For example, when considering other types of psychostimulants, two randomized controlled trials with repeated CBD administration on cocaine or crack cocaine users found no differences in cue-induced craving or relapse risk (Meneses-Gaya et al. 2020; Mongeau-Pérusse et al. 2021); conversely, controlled trials found a protective action of CBD for other drug classes (e.g. cannabinoids, opioids), either by reducing cue-induced craving or drug consumption (Freeman et al. 2020; Hurd et al. 2019; Morgan et al. 2013). For the specific case of amphetamines, there are different pathways in which CBD may have therapeutic effects for addiction-related outcomes. CBD agonist actions on serotoninergic and dopaminergic receptors could play a relevant role, given that the agonist action on 5-HT1a receptors can inhibit behaviours related to addiction, and partial dopaminergic agonists have been shown to attenuate stimulants self-administration in pre-clinical models (Calpe-López et al. 2019; Müller and Homberg 2015). Nevertheless, while much emphasis has been given on whether cannabinoids may help with problematic substance use-related craving and relapse, a main role for cannabinoid products may also be able to decrease deleterious biological impact (e.g. neuronal toxicity, psychiatric symptoms) of amphetamines and other drugs, as has been demonstrated by several of the pre-clinical studies included in this review (Castelli et al. 2014; Karimi-Haghighi et al. 2020; Prud'homme et al. 2015; Razavi et al. 2020; Valvassori et al. 2011).

The present review’s limited search and selection approach, including English-language studies only, number of databases, exclusive reliance on peer-reviewed (i.e. no grey literature) studies, may imply limitations of the present work. Furthermore, no meta-analyses of the study data were done given the heterogeneous approach and designs. In conclusion, data from pre-clinical studies suggest potentially protective effect of THC and CBD regarding amphetamine (or methamphetamine)-related addiction behaviour and impairments, while human studies, despite some preliminary protective results limited to specific populations, suggest greater morbidity between the co-use of cannabis and amphetamines in a range of (e.g. brain, cognitive, health) outcomes but mostly assessing natural cannabis use or abuse. Given recent evidence of increasing psychostimulant use and harms (e.g. in North America and elsewhere (Farrell et al. 2019; Fischer et al. 2021; Strickland et al. 2019)) and substantive pre-clinical evidence of cannabinoids therapeutic potential for addiction (Chye et al. 2019; Parsons and Hurd 2015), the dearth of systematic, rigorous (e.g. controlled clinical) studies assessing the potentially beneficial effects of cannabinoids on amphetamine-related health or behavioural outcomes should urgently be addressed.

## Supplementary Information

Below is the link to the electronic supplementary material.

Supplementary file1 (DOCX 20 KB)

## Abbreviations

AMPH: Amphetamine
aOR: Adjusted odds ratio
BDNF: Brain-derived neurotrophic factor
CBD: Cannabidiol
CB1: Cannabinoid receptor 1
CB2: Cannabinoid receptor 2
CI: Confidence interval
delta-8-THC: Delta-8-tetrahydrocannabinol
ECS: Endocannabinoid system
GFAP: Glial fibrillary acidic protein
Glu + Gln/PCr + Cr: Glutamate plus glutamine/phosphocreatine plus creatine ratio
GPC + PC/PCr + Cr: Glycerophosphocholine plus phosphocholine/phosphocreatine plus creatine ratio
i.c.: Intracerebral
i.c.v.: Intracerebral ventricular
IL-1β: Interleukin 1β
IL-6: Interleukin 6
IL-10: Interleukin 10
i.p.: Intraperitoneal
METH: Methamphetamine
mI/PCr + Cr: Myo-inositol/phosphocreatine plus creatine ratio
MRS: Proton magnetic resonance spectroscopy
mRNA: Messenger RNA
mTOR: Mammalian target of rapamycin
NSDUH: National Survey of Drug Use and Health
OR: Odds ratio
p-AKT: Phosphorylated protein kinase B
p-CREB: Phosphorylated cAMP-response element binding protein
PET: Positron emission tomography
p-GSK-3β: Phosphorylated glycogen synthase kinase 3β
PPI: Prepulse inhibition
PRESS: Peer Review Electronic Search Strategies
p70S6K: P70S6 kinase
rCMRglc: Lower cerebral glucose metabolism
s.c.: Subcutaneous
SEM: Standard error of the mean
THC: Tetrahydrocannabinol
tNAA/PCr + Cr: N-acetylaspartate plus N-acetylaspartyl glutamate/phosphocreatine plus creatine ratio
TNF-α: Tumor necrosis factor α
US: United States
5-HT: 5-Hydroxytryptamine (serotonin
