# Supplementary material for: Co-exposure of cannabinoids with amphetamines and biological, behavioural and health outcomes: a scoping review of animal and human studies
Source: Psychopharmacology (Berl). 2021 Oct 6;239(5):1211–30. doi: 10.1007/s00213-021-05960-2 (PMC9110457; doi:10.1007/s00213-021-05960-2)
Supplement: Supplementary file 1 — Supplementary file1 (DOCX 20 KB) [file 213_2021_5960_MOESM1_ESM.docx]

**Title: Co-administration of cannabinoids with amphetamines and biological, behavioural and health outcomes: a scoping review of animal and human studies**

Dimitri Daldegan-Bueno, MSc ^1^[ORCID: 0000-0002-9352-2873]

Lucas O. Maia, PhD ² [ORCID: 0000-0002-9931-1938]

Michelle Glass, PhD ^3^ [ORCID: 0000-0002-5997-6898]

Didier Jutras-Aswad, MD, MSc ^4,5^ [ORCID: 0000-0002-8474-508X]

Benedikt Fischer, PhD* ^1,2,6,7^ [ORCID: 0000-0002-2186-4030]

^1^ Schools of Population Health and Pharmacy, Faculty of Medical and Health Sciences, University of Auckland, Auckland, New Zealand (85 Park Rd, Grafton, Auckland 1023, New Zealand)

^2^ Centre for Applied Research in Mental Health & Addiction, Simon Fraser University, Vancouver, Canada (515 W. Hastings Street Vancouver, BC V6B 5K3)

^3^ Department of Pharmacology and Toxicology, University of Otago, Otago, New Zealand (PO Box 56, Dunedin 9054 New Zealand)

^4^ Centre de Recherche, Centre Hospitalier Universitaire de Universite de Montreal (CHUM), Montreal, Canada (1051 Rue Sanguinet, Montréal, QC H2X 3E4, Canada)

^5^ Department of Psychiatry and Addiction, Faculty of Medicine, Université de Montréal, Montreal, Canada (Pavillon Roger-Gaudry, 2900 Edouard Montpetit Blvd, Montreal, Quebec H3T 1J4, Canada)

^6^ Department of Psychiatry, University of Toronto, Toronto, Canada (250 College Street, 8th floor Toronto, ON. M5T 1R8)

^7^ Department of Psychiatry, Federal University of Sao Paulo (UNIFESP), Sao Paulo, Brazil (R. Dr. Ovídio Pires de Campos, 785 05403-903, Sao Paulo, Brazil)

Word Count (text body): 4,434

Submitted to: *Psychopharmacology as Review*

Submission Date: 1 March 2021

** Corresponding Author:*

Benedikt Fischer, PhD

Faculty of Medical and Health Sciences, University of Auckland,

85 Park Rd, Grafton, Auckland 1023, New Zealand
Email: bfischer@sfu.ca

Tel: +64-9-373-7599

| **Table S1.** List of terms included in the search strategy | |
| --- | --- |
| **Substance Category** | **Terms included** |
| Cannabis/Cannabinoids | cannabis; marihuana; marijuana; cannabis indica; Ganja; ganjas; hashish; hashishs; bhang; bhangs; cannabis sativa; cannabinoid; cannabinoids; cannabidiol; dronabinol; CBD; delta-9-tetrahydrocannabinol; THC; tetra-tetrahydrocannabinol |
| Amphetamines | amphetamines; amphetamine; methamphetamine |

| **Table S2.** Complete search strategies by database utilized on October 27, 2020 | | |
| --- | --- | --- |
| **Database** | **Search Strategy** | **Additional Information** |
| Medline | ((((Cannabis[Title/Abstract] OR Marihuana[Title/Abstract] OR Marijuana[Title/Abstract] OR Cannabis indica[Title/Abstract] OR Ganja[Title/Abstract] OR Ganjas[Title/Abstract] OR Hashish[Title/Abstract] OR Hashishs[Title/Abstract] OR Bhang[Title/Abstract] OR Bhangs[Title/Abstract] OR Cannabis sativa [Title/Abstract] OR Cannabinoid[Title/Abstract] OR Cannabinoids[Title/Abstract] OR Cannabidiol[Title/Abstract] OR Dronabinol[Title/Abstract] OR CBD [Title/Abstract] OR delta-9-tetrahydrocannabinol[Title/Abstract] OR THC[Title/Abstract] OR tetra-tetrahydrocannabinol[Title/Abstract]) AND (Amphetamines[Title/Abstract] OR Amphetamine[Title/Abstract] OR Methamphetamine[Title/Abstract])) AND (("2000/01/01"[Date - Publication] : "3000"[Date - Publication]))) AND (English[Language])) AND (Journal Article[Publication Type]) | Interface: Medline |
| Web of Science (Core Collection) | (TS=(Amphetamines OR Amphetamine OR Methamphetamine)) AND (TS=(Cannabis OR Marihuana OR Marijuana OR Cannabis indica OR Ganja OR Ganjas OR Hashish OR Hashishs OR Bhang OR Bhangs OR Cannabis sativa OR Cannabinoid OR Cannabinoids OR Cannabidiol OR Dronabinol OR CBD OR delta-9-tetrahydrocannabinol OR THC OR tetra-tetrahydrocannabinol) ) AND (PY=(2000-2020)) | Interface: Clarivate Analytics  Language (English) and document type (Article OR Data Paper) were manually selected |
| PsycInfo | ((Cannabis or Marihuana or Marijuana or Cannabis indica or Ganja or Ganjas or Hashish or Hashishs or Bhang or Bhangs or Cannabis sativa or Cannabinoid or Cannabinoids or Cannabidiol or Dronabinol or CBD or delta-9-tetrahydrocannabinol or THC or tetra-tetrahydrocannabinol) and (Amphetamines or Amphetamine or Methamphetamine)).tw. and ("2000" or "2001" or "2002" or "2003" or "2004" or "2005" or "2006" or "2007" or "2008" or "2009" or "2010" or "2011" or "2012" or "2013" or "2014" or "2015" or "2016" or "2017" or "2018" or "2019" or "2020").yr. and English.lg. and Peer-Reviewed Journal.pt. | Interface: Ovid |
| CINAHL Plus | (Cannabis OR Marihuana OR Marijuana OR Cannabis indica OR Ganja OR Ganjas OR Hashish OR Hashishs OR Bhang OR Bhangs OR Cannabis sativa OR Cannabinoid OR Cannabinoids OR Cannabidiol OR Dronabinol OR CBD OR delta-9-tetrahydrocannabinol OR THC OR tetra-tetrahydrocannabinol ) AND ( Amphetamines OR Amphetamine OR Methamphetamine) AND LA English AND PT ( Systematic Review OR Meta Analysis OR Randomized Controlled Trial OR Research OR Review ) AND PY 2000-2020  Amphetamines OR Amphetamine OR Methamphetamine | Interface: EBSCOhost Research Databases |
|  | | |
